# Preoperative serum uric acid predicts incident acute kidney injury following cardiac surgery

**DOI:** 10.1186/s12882-018-0970-x

**Published:** 2018-07-04

**Authors:** T. Kaufeld, K. A. Foerster, T. Schilling, J. T. Kielstein, J. Kaufeld, M. Shrestha, H. G. Haller, A. Haverich, B. M. W. Schmidt

**Affiliations:** 1 Department of Heart, Thoracic, Transplant and Vascular Surgery, Hannover Medical School, Carl-Neuberg-Straße 1, 30625 Hannover, Germany; 2 Department of Nephrology and Hypertension, Hannover Medical School, Carl-Neuberg-Str.1, Hannover, 30625 Germany

**Keywords:** Renal impairment, Cardiac surgery, Serum uric acid, Kidney injury

## Abstract

**Background:**

Acute kidney injury (AKI) following cardiac surgery is a frequent complication and several risk factors increasing its incidence have already been characterized. This study evaluates the influence of preoperative increased serum uric acid (SUA) levels in comparison with other known risk factors on the incidence of AKI following cardiac surgery.

**Methods:**

During a period of 5 month, 247 patients underwent elective coronary artery bypass grafting, valve replacement/ repair or combined bypass and valve surgery. Datas were prospectively analyzed. Primary endpoint was the incidence of AKI as defined by the AKI criteria comparing patients with preoperative serum uric acid (SUA) levels below versus above the median. Multivariate logistic regression analysis was used to identify independent predictors of postoperative AKI.

**Results:**

Thirty (12.1%) of the 247 patients developed postoperative AKI, 24 of 30 (80%) had preoperative SUA- levels above the median (≥373 μmol/l) (OR: 4.680, CI 95% 1.840; 11.904, *p* = 0.001). In the multivariate analysis SUA levels above the median (OR: 5.497, CI 95% 1.772; 17.054, *p* = 0.003), cardiopulmonary bypass (CPB) time > 90 min (OR: 4.595, CI 95% 1.587; 13.305, *p* = 0.005), cardiopulmonary bypass (CPB) > 30 kg/m^2^ (OR: 3.208, CI 95% 1.202; 8.562; *p* = 0.02), and preoperative elevated serum-creatinine levels (OR: 1.015, CI 95% 1.001; 1.029, *p* = 0.04) were independently associated with postoperative AKI.

**Conclusions:**

Serum uric acid is an independent risk marker for AKI after cardiac surgery. From all evaluated factors it showed the highest odds ratio.

## Background

Acute kidney injury (AKI) following cardiac surgery is a frequent complication and has a significant impact on postoperative mortality [1–5]: overall mortality after open-heart surgery ranges between 2 and 8% [5] and raises up to 29% in patients developing postoperative AKI and even over 60% in those requiring postoperative renal replacement therapy [4].

Risk factors increasing the incidence of AKI after cardiac surgery imply age [4, 6, 7], female gender [1, 6–8] and several comorbidities as hypertension [9], peripheral vascular disease [1, 2, 4, 6], diabetes mellitus [1, 2, 6, 7, 9], congestive heart failure [1, 2, 4], chronic obstructive pulmonary disease [1, 2, 4], prior heart surgery, recent myocardial infarction (< 7–30 days) [2, 4, 6, 7, 10], preoperative creatinine concentrations > 1.3 mg/dl and current diuretic use [2, 9, 11]. The incidence of AKI is associated with the type of operation: combined surgery (coronary artery bypass grafting plus valvular heart surgery) implies a higher risk for AKI than each procedure by itself [3, 4]. Valvular heart surgery is described as a single independent risk factor [1, 4, 12], especially mitral valve replacement or repair [7]. Risk factors concerning the operation itself imply the duration of aortic cross clamp [11], need for emergency surgery [1, 6], reoperation for bleeding or repeat cardiopulmonary bypass [10], intra-aortic balloon pump (IABP) insertion [6] and number of intraoperative packed red blood cell (PRBC) units transfused [11]. The duration of the on-pump cardiopulmonary bypass (CPB) is viewed as one of the most important predictive marker for the development of postoperative AKI [1, 6, 7, 13, 14].

Ejaz et al. [15] have examined the role of serum uric acid (SUA) as a potential risk factor for AKI after cardiac valve and aneurysm surgery. Preoperative SUA higher than an arbitrary level of 6.1 mg/dl conferred a 4-fold risk for postoperative AKI.

AKI induced by increased uric acid levels commonly occurs in patients with tumor lysis syndrome (TLS). In this context uric acid impairs renal function by intratubular crystal precipitation as well as by inducing oxidative stress and renal inflammation [16]. Kuwabara et al. [17] as well as Feig et al. [18] hypothesized that elevated uric acid levels have a role in kidney disease: they impair endothelial function and cause subtle renal damage.

With regard to Feig et al. [18] and in view of the fact that AKI following cardiac surgery is a frequent complication we performed a study to determine if SUA is an independent predictive marker for AKI in patients undergoing cardiac surgery.

## Methods

In a prospective study we analyzed 247 patients who underwent elective cardiac surgery during a period of 5 months at the Medizinische Hochschule Hannover (MHH), Germany. The procedures were coronary artery bypass grafting (CABG) in 109 patients, valve replacement or repair (VR) in 93 patients, combined heart surgery (CABG + VR) in 44 patients and resection of left ventricular aneurysm (other) in one patient. Concomitant procedures were the replacement of the aortic root or the aorta ascendens in 27 patients, operation of the carotid artery in 4 patients, insertion of an intra aortic balloon pump (IABP) in 3 patients and the operation of the femoral artery in one patient. The study received a waiver by the Institutional Review Board of the Medizinische Hochschule Hannover (MHH).

Primary endpoint of the study is the incidence of AKI as defined by the AKIN criteria [19, 20] comparing patients with preoperative serum uric acid (SUA) levels below versus above the median. Patients with preoperative chronic kidney disease stage 5 (estimated glomerular filtration rate at baseline < 15 ml/min or on permanent renal replacement therapy), preoperative intensive care unit stay > 24 h and emergency surgery were not included in this study.

We collected data including demographic variables, type of surgery, comorbidities and preoperative medication. Preoperative SUA and serum creatinine (SCr) levels were measured in the context of the preoperative routine laboratory assessment. Univariate analysis was performed to determine the association between preoperative SUA levels above the median, demographic parameters, surgery type, renal function, comorbidities, preoperative medication including allopurinol use, intraoperative parameters, and the incidence of AKI. We used X^2^- test and two-sided Fisher’s exact test as appropriated. *P*-value < 0.05 was considered statistically significant.

Binary logistic regression analysis was performed to evaluate if SUA levels above the median are an independent marker for developing AKI following cardiac surgery and to determine further independent risk factors. The following variables showed a *p*-value < 0.2 in univariate analysis and entered into the logistic regression analysis: preoperative SUA > median, body mass index (BMI) > 30 kg/m^2^, valve replacement or repair (VR), coronary artery bypass grafting (CABG), preoperative serum creatinine (SCr) > median, diabetes, preoperative diuretic use and cardiopulmonary bypass (CPB) time > 90 min. Referring to previous studies we chose CPB- time > 90 min as the strongest representative factor for operations’ severity [1, 6, 7, 13, 14]. Therefore operation- time, aortic cross clamp (ACC) time, need for reoperation and numbers of intraoperative transfused FFP did not enter into logistic regression analysis. *P*-value < 0.05 was considered statistically significant.

Statistical analysis was performed using SPSS, Version 19, IBM Germany, Ehningen.

## Results

Demographic data are shown in Table 1. Preoperative SUA levels above the median (≥373 μmol/l) were significantly associated with higher serum creatinine and GFR, more diuretic and less allopurinol use.

**Table 1:** Baseline patient characteristics

Variables	Uric acid > median (*n* = 124)	Uric acid < median (*n* = 123)	*P*- Value
Serum uric acid (μmol/l; mean ± SD)	464.94 ± 91.066	301.93 ± 45.434	
Demographics			
Age (yr; mean ± SD)	67.40 ± 12.221	68.26 ± 10.851	0.557
Female gender (*n*; %)	34 (27.4)	48 (39.0)	0.053
BMI (mean ± SD)	28.213 ± 4.4752	27.089 ± 4.5357	0.054
Surgery type: *n* (%)			
Valves	43 (34.7)	50 (40.7)	0.335
Bypass	59 (47.6)	50 (40.7)	0.275
Valves + Bypass	22 (17.7)	22 (17.9)	0.976
Other	−	1 (0.8)	
Renal function			
Serum- Creatinine (μmol/l; mean ± SD)	101.35 ± 31.794	81.89 ± 26.336	< 0.001
CKD- Epi- GFR (mean ± SD)	66.865 ± 22.0789	78.676 ± 18.1805	< 0.001
Comorbidities: *n* (%)			
Hypertension	114 (91.9)	110 (89.4)	0.264
CKD	38 (30.6)	17 (13.8)	0.001
Diabetes	41 (33.1)	27 (21.0)	0.050
PVD	17 (13.7)	13 (10.6)	0.452
Congestive heart failure	94 (75.8)	89 (72.4)	0.332
COPD	19 (15.3)	13 (10.6)	0.257
Previous heart surgery	16 (12.9)	9 (7.3)	0.130
Medication: *n* (%)			
ACE- Inhibitor	67 (54.0)	62 (50.4)	0.250
AT1- Receptor- Blocker	16 (12.9)	17 (13.8)	0.984
Calcium Antagonist	27 (21.8)	28 (22.8)	0.940
Βeta- Blocker	76 (61.3)	73 (59.3)	0.371
Diuretic	56 (45.2)	43 (35.0)	0.034
Lipid- lowering drugs	51 (41.1)	54 (43.9)	0.983
Allopurinol	3 (2.4)	12 (9.8)	0.020
Intraoperative parameters			
Operation time (minutes; mean ± SD)	213.26 ± 85.004	203.81 ± 81.271	0.373
CPB- time (minutes; mean ± SD)	104.08 ± 67.311	103.31 ± 64.880	0.928
ACC- time (minutes; mean ± SD)	60.53 ± 40.365	61.31 ± 36.619	0.876
PRBC (mean ± SD)	2.30 ± 3.193	1.98 ± 2.054	0.343
FFP (mean ± SD)	1.44 ± 2.470	0.96 ± 1.822	0.084
Need for reoperation (*n*; %)	17 (13.7)	16 (13.0)	0.872

*BMI* Body mass index, *CKD- Epi- GFR* Chronic Kidney Disease Epidemiology Collaboration Glomerular Filtration Rate, *CKD* Chronic Kidney Disease, *PVD* Peripheral Vascular Disease, *COPD* Chronic Obstructive Pulmonary Disease, *ACE*  Angiotensin- converting Enzyme, *AT1- Receptor- Blocker* Angiotensin- 1- Receptor- Blocker, *CPB* Cardiopulmonary Bypass, *ACC* Aortic Cross Clamp, *PRBC* Packed Red Blood Cells, *FFP* Fresh Frozen Plasma

Thirty (12.1%) of 247 patients developed AKI following cardiac surgery. 24 (80%) of 30 patients that developed postoperative AKI had preoperative SUA levels above the median (OR: 4.680, CI 95% 1.840; 11.904, *p* = 0.001, Table 2). 8 (3.2%) of 247 patients required postoperative renal replacement therapy, 7 (87.5%) of 8 patients had SUA levels above the median (OR: 7.299, CI 95% 0.884; 60.240, *p* = 0.066). There was no intrahospital mortality.

**Table 2:** Univariate analysis of predictors of AKI

Variables	Odds ratio (CI 95%)	*P*-value
Serum uric acid > median	4680 (1.840; 11.904)	0.001
Demographics		
Age > 60 yr	1.749 0.581; 5.263	0.467
Male gender	0.840 (0.379; 1.860)	0.682
BMI > 30 kg/m^2^	3.220 (1.472; 7.045)	0.004
Surgery type: n (%)		
Valves	1.782 (0.827; 3.839)	0.161
Bypass	0.418 (0.178; 0.979)	0.049
Valves + Bypass	1.481 (0.592; 3.704)	0.444
Renal function		
Serum- Creatinine > median	4.509 (1.967; 10.338)	< 0.001
CKD- Epi- GFR < 60 ml/min	2.875 (1.321; 6.257)	0.009
Comorbidities: n (%)		
Hypertension	1.357 (0.300; 6.143)	1.000
CKD	3.938 (1.763; 8.800)	0.001
Diabetes	2.609 (1.195; 5.695)	0.017
PVD	1.990 (0.739; 5.355)	0.228
Congestive heart failure	1.553 (0.563; 4.284)	0.488
COPD	1.896 (0.706; 5.091)	0.237
Previous heart surgery	3.280 (1.238; 8.689)	0.021
Medication: n (%)		
ACE- Inhibitor	1.131 (0.503; 2.544)	0.839
AT1- Receptor- Blocker	1.289 (0.452; 3.671)	0.579
Calcium Antagonist	2.206 (0.962; 5.056)	0.064
Βeta- Blocker	1.260 (0.526; 3.014)	0.672
Diuretic	1.783 (0.801; 3.971)	0.161
Lipid- lowering drugs	0.688 (0.307; 1.546)	0.421
Allopurinol	0.476 (0.060; 3.769)	0.700
Intraoperative parameters		
Operation time > 300 min	4.466 (1.795; 11.115)	0.002
CPB- time > 90 min	4.360 (1.854; 10.252)	0.001
ACC- time > median	2.502 (1.124; 5.572)	0.036
PRBC transfusion	0.710 (0.327; 1.540)	0.420
FFP transfusion	2.776 (1.279; 6.024)	0.012
Need for reoperation	5.132 (2.164; 12.170)	< 0.001

*BMI* Body mass index, *CKD- Epi- GFR* Chronic Kidney Disease Epidemiology Collaboration Glomerular Filtration Rate, *CKD* Chronic Kidney Disease, *PVD* Peripheral Vascular Disease, *COPD* Chronic Obstructive Pulmonary Disease, *ACE* Angiotensin- converting Enzyme; *AT1- Receptor- Blocker* Angiotensin- 1- Receptor- Blocker, *CPB* Cardiopulmonary Bypass, *ACC* Aortic Cross Clamp, *PRBC* Packed Red Blood Cells, *FFP* Fresh Frozen Plasma

In univariate analysis a BMI > 30 kg/m^2^ (OR: 3.220, CI 95% 1.472; 7.045, *p* = 0.004) and CABG (OR: 0.418, CI 95% 0.178; 0.979, *p* = 0.049), preoperative serum creatinine (OR: 4.509, CI 95% 1.967; 10.338, *p* < 0.001), chronic kidney disease (OR: 3.938, CI 95% 1.763; 8.800, *p* = 0.001) and diabetes (OR: 2.609, CI 95% 1.195; 5.695, *p* = 0.017) were significantly associated with the incidence of AKI. Intraoperative parameters as operation time > 300 min (OR: 4.466, CI 95% 1.795; 11.115, *p* = 0.002), cardiopulmonary bypass (CPB) time > 90 min (OR: 4.360, CI 95% 1.854; 10.252, p = 0.001), aortic cross clamp (ACC) time above the median (OR: 2.502, CI 95% 1.124; 5.572, *p* = 0.036) and the number of intraoperative transfused fresh frozen plasma (FFP) (OR: 2.776, CI 95% 1.279; 6.024, *p* = 0.012) go along with a higher risk for postoperative AKI.

After adjusting for BMI, CABG, VR, CPB > 90 min, preoperative SCr, diabetes and preoperative diuretic use the following variables were independently associated with AKI: SUA levels above the median (OR: 5.497, CI 95% 1.772; 17.054, *p* = 0.003), BMI > 30 kg/m^2^ (OR: 3.208, CI 95% 1.202; 8.562; *p* = 0.02), CPB time > 90 min (OR: 4.595, CI 95% 1.587; 13.305, *p* = 0.005) and preoperative elevated SCr levels (OR: 1.015, CI 95% 1.001; 1.029, *p* = 0.04). In contrast, type of surgery (CABG, VR), diabetes and preoperative diuretic drug use were not independently associated with the incidence of AKI following cardiac surgery in logistic regression analysis. The results of binary logistic regression analysis are presented in Table 3.

**Table 3:** Multivariable analysis of predictors of AKI

Variables	Odds ratio (CI 95%)	*P*- Value
Serum uric acid > median	7131 (2.061; 24.667)	0.002
BMI > 30 kg/m^2^	3.158 (1.102; 8.468)	0.020
Valves	0.363 (0.094; 1.399)	0.141
Bypass	1.016 (0.236; 4.387)	0.983
Diabetes	0.452 (0.147; 1.389)	0.166
Diuretic	0.988 (0.356; 2.118)	0.769
Calcium Antagonist	1.864 (0.628; 5.537)	0.262
CPB- time > 90 min	4.337 (1.478; 12.304)	0.018
Serum Creatinine	0.984 (0.969; 0.999)	0.032
FFP Transfusion	1.007 (0.795; 1.277)	0.952
Need for Reoperation	7.154 (1.917; 26.699)	0.003
Previous Heart Surgery	2.205 (0.581;8.375)	0.245

*BMI* Body mass index, *CPB* Cardiopulmonary bypass

## Discussion

This study evaluates the influence of preoperative increased serum uric acid (SUA) levels and further concomitant factors on the incidence of AKI following cardiac surgery. We detected SUA as an independent and strong predictive marker for developing AKI after VR, CABG and VR plus CABG. BMI > 30 kg/m^2^, preoperative elevated serum creatinine (SCr) levels and CPB duration > 90 min are as well independently associated with a higher incidence of postoperative AKI.

The acute tubular necrosis (ATN) as the histopathological correlate of postoperative AKI results from reduced renal perfusion by intraoperative impaired hemodynamics and low cardiac output [1, 4, 21]. A longer duration of CPB goes along with higher risk for renal ischemia and causes renal inflammation by inducing an increase in cytokines such as TNF-α, IL-1 and IL-6. Moreover CPB can cause episodic microembolism leading to renal infarction. [1, 4, 14].

In patients with tumor lysis syndrome (TLS) increased uric acid induces AKI by intratubular crystal precipitation as well as on a crystal- independent pathway [16]. By stimulating the renin- angiotensin system, reducing nitric oxide (NO) release from endothelial cells and inhibiting NO synthase 1 uric acid causes renal vasoconstriction and leads to renal ischemia and hypertension [22]. Uric acid is also considered to have proinflammatory properties: in-vitro it induced the expression of C-reactive protein (CRP) by human endothelial and vascular smooth muscle cells and the production of the monocyte chemoattractant protein-1 (MCP-1) [23, 24]. By stimulating the proliferation of vascular smooth muscle cells while inhibiting endothelial cell growth at the same time, uric acid impairs renal autoregulation and reduces the GFR [22, 25]. Those mechanisms result in glomerulosclerosis, interstitial fibrosis and arteriolar disease [18].

So far clinical and experimental studies have demonstrated that increased SUA can cause AKI and can induce progression of chronic kidney disease in a non- operative setting [26–28]. Ejaz et al. [15] offered preliminary indication that uric acid impacts the development of AKI following cardiac surgery. By regarding a small study population of 58 patients increased preoperative SUA levels above an arbitrary level of 6.1 mg/dl determined a 4-fold risk for AKI. Common procedures as CABG and CABG plus VR were not examined in this study. Talwar et al. [29] found that preoperative allopurinol treatment in patients undergoing valvular heart surgery is associated with decreased postoperative inotropic requirement and duration of postoperative mechanical ventilation and shorter hospital stay.

According to several studies we could verify CPB time as an independent intraoperative risk factor for AKI following cardiac surgery [1, 6, 7, 13, 14]. Chertow et al. [2] have already detected preoperative increased SCr as an important marker for postoperative AKI. Although the incidence of AKI after cardiac surgery is described as more frequent in valvular heart surgery or in CABG plus VR [1, 3, 4, 12], we could not verify the association of postoperative AKI with the type of surgery.

## Conclusion

In conclusion, we assume that hyperuricemia could lead to AKI by two different pathways: chronically it may have induced subtle renal damage that predisposes to AKI and that may not be preoperatively detected by serum creatinine. Acutely the effect of intraoperative renal hypoperfusion may be aggravated by the proinflammatory and prooxidative properties of SUA.

Finally, our data indicates that measuring of SUA levels may contribute to preoperative risk assessment. Inhibition of xanthin oxidase should be evaluated in prospective randomised controlled trials.

## Abbreviations

ACC: Aortic cross clamp
AKI: Acute kidney injury
ATN: Acute tubular necrosis
ATN: Acute tubular necrosis
CPB: Cardiopulmonary bypass
FFP: Fresh frozen plasma
IABP: Intra-aortic balloon pump
MCP-1: Monocyte chemoattractant protein-1
MHH: Medizinische Hochschule Hannover
NO: Nitric oxide
OR: Odds ratio
PRBC: Packed red blood cell
SCr: Serum creatinine
SUA: Serum uric acid
TLS: Tumor lysis syndrome
VR: Valve replacement/repair
